# Polymeric Nanoscale Drug Carriers Mediate the Delivery of Methotrexate for Developing Therapeutic Interventions Against Cancer and Rheumatoid Arthritis

**DOI:** 10.3389/fonc.2020.01734

**Published:** 2020-09-16

**Authors:** Wen-Jun Yu, Dong-Xu Huang, Shuang Liu, Ying-Li Sha, Feng-hui Gao, Hong Liu

**Affiliations:** ^1^The Eastern Division, Department of Hand and Foot Surgery, The First Hospital of Jilin University, Changchun, China; ^2^The Eastern Division, Department of Nursing Management, The First Hospital of Jilin University, Changchun, China; ^3^The Eastern Division, Department of Pediatrics, The First Hospital of Jilin University, Changchun, China; ^4^The Eastern Division, Department of Orthopaedics, The First Hospital of Jilin University, Changchun, China; ^5^The Eastern Division, Department of Otolaryngology, The First Hospital of Jilin University, Changchun, China

**Keywords:** methotrexate, cancer, liposomes, LPHNPs, NLCs

## Abstract

Methotrexate (MTX) is widely used as an anticancer and anti-inflammtory drug for treating various types of cancer and autoimmune diseases. The optimal dose of MTX is known to inhibit the dihydrofolatereductase that hinders the replication of purines. The nanobiomedicine has been extensively explored in the past decade to develop myriad functional nanostructures to facilitate the delivery of therapeutic agents for various medical applications. This review is focused on understanding the design and development of MTX-loaded nanoparticles alongside the inclusion of recent findings for the treatment of cancers. In this paper, we have made a coordinated effort to show the potential of novel drug delivery systems by achieving effective and target-specific delivery of methotrexate.

## Introduction

The aim of achieving the utmost therapeutic efficacy with the fewest drug hazards is always a priority for any pharmaceutical researcher. The available therapeutic options, such as chemotherapy and radiotherapy, need skilled personnel to lead to better results from the target-specificity of the drug(s) in question. The traditional drug delivery systems earned popularity due to their economic, simple, and user-friendly approach, but recently developed specific drug-delivery systems, such as lipid-polymer hybrid nanoparticles (1), have drawn attention due to their target-specificity, effectiveness, and fewer adverse effects.

Methotrexate (MTX) (also known as amethoptrein; MW: 454 g/mol) is a widely used drug for multiple medical conditions, such as psoriasis, rheumatoid arthritis (RA), and cancer (2). This drug is also approved for the treatment of Crohn's disease by the U.S. Foog and Drug Administration (3). MTX (2,4-diamino-N10-methyl propylglutamic acid) was first synthesized by Seeger et al. nearly 65 years ago (4). Its structure encompasses three parts: (1) a pteridine ring, (2) p-aminobenzoic acid, and (3) glutamic acid (4). It is a weak, pH-dependent bicarboxylic acid having pKa values of 3.8, 4.8, and 5.6 with low permeability (log *p* = 0.53) (5). It is heat- and light-sensitive and degrades upon exposure, and its solubility in distilled water at 20°C is 0.01 mg/ml; suitable pH for MTX falls in the range of 6.6–8.2 (6).

Methotrexate blocks the activity of the dihydrofolatereductase (DHFR) enzyme and leads to the inhibition of DNA synthesis at higher dosages (2). MTX is not considered to be an antiproliferative agent in the inflammatory joints during RA pathology. However, lower dosages of MTX and its discontinuation show an anti-inflammatory effect of MTX (4). MTX-mediated inhibition of DHFR and other folate-dependent enzymes leads to the overproduction of adenosine, which drives immunosuppression (7). Despite initial obstacles to the use of novel drug-delivery systems (NDDS), the nanotechnology helps in achieving maximum drug therapeutics. Nanotechnology is seen as a promising strategy for the treatment of various medical conditions by active and passive targeting (8). The effectiveness of treatment is associated with the ability of a drug to target and affect the biological functions of ailing cells, leaving minimal damage to healthy tissues (8). Nanoparticles take advantage of unique characteristics, such as the enhanced permeation and retention (EPR) effect, a large surface-to-volume ratio, extended residence time in circulation, biodegradability, low toxicity, and small size in the range of 10–500 nm, thus conferring sustained and targeted drug delivery (9, 10). Efforts have been made to develop nanodrug delivery vehicles, including polymeric nanoparticles (PNPs) (11), lipid-polymer hybrid nanoparticles (LPHNPs) (12), nanostructured lipid carriers (NLCs) (13), solid lipid nanoparticles (SLNs) (14), and liposomes (15) for the controlled and targeted delivery of MTX. This paper reviews the development of surface-engineered, lipid-based nanocarriers (SLNs and LPHNPs), which are proposed to improve the delivery of drugs.

In the end, this review has been designed to explore the applications of MTX in different clinical settings with cancer. We discuss the role of NDDS to find out the solutions by improvising the treatment strategies. We believe this review is a compilation of our concerted efforts to cover all aspects and dimensions of drug delivery.

## Mechanism of Action of MTX and Clinical Pharmacology

### Clinical Pharmacodynamic

MTX inhibits the DHFR enzyme, which is required to reduce dihydrofolates to tetrahydrofolates before they are utilized as carbon carriers during purine nucleotide synthesis. Therefore, MTX hinders the synthesis, repair, and cellular replication of DNA (16). In addition to the abovementioned action for the clinical efficacy of MTX, several other interlinked biochemical mechanisms are involved, substantiating its usefulness in the treatment of other diseases, such as neoplastic diseases, psoriasis, and adult RA (7). MTX is more sensitive to actively proliferating cells, such as malignant cells, fetal cells, bone marrow, buccal and intestinal mucosa, and urinary bladder cells (2). MTX impairs malignant growth without irreversible damage to the normal tissues during cellular proliferation in which malignant tissues outgrow the normal tissues. The wider range of applications and selective action of MTX proves it to be an efficacious therapeutic drug (3, 4), and therefore, its pharmacology is extensively studied (13).

### Clinical Pharmacokinetics

The pharmacokinetics of MTX were performed by various techniques (bacteriological assay followed by fluorometric assay). Currently, it is mainly measured in biological fluids by high-performance liquid chromatography or fluorescence polarization immunoassay (FPI) (2). Currently, FPI is in use for the measurement of plasma concentration when a high dose of MTX (> 1 g/m^2^) with the low limit quantitation (0.02 μM or 9 μg/l) is employed. The bioavailability of MTX delivered through different routes of administration is accounted and described below:

#### Oral Absorption

A high dose (≤ 25 mg) of MTX is generally administered through the oral route in a week. This was found to be dose-dependent, incomplete, and highly variable (absolute bioavailability range: 13% − 76%) (17). It has been observed that oral absorption is better at a dose of <40 mg/m^2^ (median bioavailability: 42%), and for dosage more than 40 mg (median bioavailability: 18%), use of the intravenous (IV) route is generally recommended. Moreover, oral administration of MTX (7.5 mg) is not influenced by food in healthy volunteers (17).

#### Subcutaneous (SC) Absorption

This is an alternative to the oral route as the drug is completely absorbed (MTX; 40 mg/m^2^) compared to that seen with the IV route when injecting in acute lymphoblastic leukemia children. C_max_ was found to be 7.4 and 1.4 μM for subcutaneous and intravenous routes, respectively (18).

#### Intramuscular (IM) Absorption

This is an alternative to the oral route for achieving low-dose administration. The bioavailability of MTX when delivered through the IM route is found to be 76%, which falls in the range between the SC and oral routes (17). Further, it is also used off-label in the treatment of tubal ectopic pregnancy. It is administered as a 1 mg/kg or 50 mg/m^2^ formulation in a single- or multiple-dose regimen (19).

#### Intrathecal Absorption

This is used in some local treatment of hematological disease via systemic diffusion of MTX after regional administration at very low doses (6–15 mg) (20).

#### Distribution

Approximately 46% of MTX binds to human serum albumin. It is given as a prophylactic or curative treatment via the intrathecal route in combination with systemic treatment with a fixed dosage between 6 and 15 mg depending upon age (20). Generally, it is administered at higher IV dosage during primary or secondary CNS treatment. The penetration of the drug into the cerebrospinal fluid is less but clinically sufficient, and it does not depend on the administered dose (2 or 5 g/m^2^) (21).

#### Metabolism and Elimination

MTX is rapidly eliminated from the human body through the renal route (90% of an intravenously injected dose is excreted in 24 h and 95% in 30 h) after being metabolized into 7-hydroxy MTX. The aldehyde oxidase mediates the biotransformation, and the metabolite is found in blood, urine, and bile due to partial intestinal reabsorption; 1–2% of the drug is also found in stool samples of patients having an intravenously administered dose in the form of the parent drug and metabolites. It is known to have a terminal half-life of 8–15 h (22). The derivatives of MTX include 7-hydroxy MTX and 2,4-diamino-N10-methylpetroic acid, which have a similar half-life of 10.2 and 9 h, respectively. Once it enters the body, irrespective of the route, mean clearance is found to be 50–135 ml/min/m^2^ (23). In a study of leukemic children receiving 1 g/m^2^ MTX, a similar pattern of clearance was observed for 1–24 h (11 and 123 ml/min/m^2^, respectively).

Recently, various molecular determinants of MTX (drug metabolizing enzymes, transporter) have been discovered, and they are involved in the pharmacokinetic process to prevent drug interactions and understand their disposition. The membrane transporters OATP1B1, OATP1B3, MRP2, MRP3, MRP4, BCRP, and RFC regulate hepatic clearance, whereas OAT1, OAT3, MRP2, MRP4, BCRP, and RFC are involved in renal elimination (2).

## Rationale of Using MTX-loaded Delivery System

Despite being a widely used drug for the treatment of tumors and autoimmune disorders, the suboptimal pharmacological response of MTX limits its use (4).

### Adverse Effects of MTX

The commonly noticed adverse effects of MTX are vomiting, nausea, anemia, diarrhea, dermatitis, bruising, hepatitis, pulmonary fibrosis, and bone marrow depression (4). MTX produces dose- and duration-dependent teratogenicity (24). MTX is not recommended for pregnant and breast-feeding women as it causes severe fetal defects, mainly neural tube defects (25), because of its teratogenic nature. Also, it affects the process of spermatogenesis, altering male fertility and producing congenital defects at 6–8 week of gestation (26). MTX could be iatrogenic because four cases of medical malpractice were reported in China due to overdose of MTX, including 10 (two cases), 15 (one case), and 20 mg (one case) per day rather than the weekly recommended dosage, and they led to mucositis and death (27). High and low doses of MTX may cause severe complications: a high dose (>1 g/m^2^) of MTX may result in kidney injury due to the crystallization of drugs or their derivatives inside the nephrons, prompting delays in renal elimination and rendering systemic toxicity (28). The delayed elimination has resulted in ≥grade 2 nephrotoxicity in 1.8 and 9.1% of osteosarcoma and lymphoma (elder) patients, respectively (2). There could be interindividual variability between 30 and 90% in peak levels, duration to achieve peak time, dose absorbance rate, and area under the serum concentration–time curve (29). MTX dose also plays a crucial role in the bioavailability of MTX as the higher dose is quickly eliminated by the kidneys, thus conferring its short half-life (5–8 h). Moreover, target specificity and drug efficacy are issues faced due to the administration of lower doses (4). The pharmacokinetics of MTX mainly depend on the route of administration when measuring the level of MTX in CSF and blood in rats. The low plasma levels in intranasally administered animals were comparable to those seen with the intravenous route, and greater MTX concentration was quantified in animals administered the drug through the intranasal route compared to the intravenous route (30). MTX was injected in a rodent animal model through transcutaneous puncture at the level of the cisterna magna, and it shows the cognitive and neurotoxic effects. In spite of the reduction seen in the folate levels in CSF and serum, a higher amount of homocystine was quantified, which supports the intrathecal delivery of MTX (31). Choudhary et al. administered MTX by the intraperitoneal route in mouse bone marrow with three different dosages (2, 10, and 20 mg/kg). It was found to be brutally effective in male mice compared to female mice. The intermediate dose of 10 mg/kg was found to be effective out of the concentrations tested (32). The use of implantable calcium phosphate systems in rabbits showed the extended release of MTX due to its adsorption on deficient apatite and favors enhanced antirheumatic activity (33).

### Targeted and Controlled Drug Delivery System

The difference in t_1/2_ needs a continuous dose of MTX to achieve optimal bioactivity within its therapeutic range as its cytotoxicity directly depends upon the mean residence time in plasma (3). For the controlled release of the drug, an encapsulated lipid-based delivery system was developed for cutaneous administration of MTX, and it enhanced plasma t_1/2_ from 0.53–100 h (190 times), and lowered C_max_ (120 times) with 130 times higher efficacy against L1210 leukemia cells (34) was estimated. Likewise, with intracavitary administration, t_1/2_ was reached in 39.6 h (encapsulated MTX) from 0.5 (unencapsulated MTX), and another lipid-based formulation injected via the intracisternal route was increased up to 5.4 days (encapsulated) from 0.30 (unencapsulated) (3). In addition, chitosan microspheres (35) and water-in-oil microemulsion (36) delivered MTX within the therapeutic range, and the inhibition of tumor growth was observed by extending apoptosis.

Therefore, the route of MTX may be an alternative approach, but the patient specificity might not work for all patients. The cause of side effects still exists irrespective of the route of drug administration. The controlled and targeted delivery approaches may have overcome the repetitive administration of MTX, but none of them are target-specific. Recently, many studies have been carried out to overcome the limitations of different NDDS. These NDDS provide better results in terms of safety, efficacy, target-specificity, improved bioavailability, and sustained drug release with higher stability of the therapeutic effect against various biochemical mechanisms. We discuss various drug delivery systems employed in cancer, RA, and psoriasis for MTX therapeutics.

### Role of MTX in Cancer Therapeutics

#### Pathophysiology of Cancer

Cancer is the second leading cause of death around the globe (37), and according to global cancer statistics in 2018 (GLOBOCAN), there are 18.1 million new cancer cases with a death toll of 9.6 million (excluding data on non-melanoma skin cancer). Lung cancer is the most commonly diagnosed type of cancer (11.6% of the total) and the leading cause of cancer death (18.4% of total deaths) followed by female breast cancer (11.6%) (by combining both genders) (38). Cancer occurs due to interruption in the routine signal transduction mechanism mediated by a normal cell, and more than 277 types of cancers are diagnosed (37). It is mainly afflicted due to the specific DNA damage mediating several mechanisms, such as activation of proto-oncogenes by translocation or by point mutation and inactivation of a gene resulting in tumor formation (39). Chemical compounds also play a role in gene mutation, including smoking and environmental chemical substances (directly/indirectly influence the cytoplasm and nucleus and leads to the gene defect/disorder/mutation) (37). There are other carcinogenic factors, such as bacteria, viruses, and radiation responsible for around 7% of total cancers (40). Cancer disturbs the cellular mechanism and, thus, leads to inappropriate function of a gene, affecting the cell cycle and abnormal proliferation. Proto-oncogenes responsible for cell growth and division are converted into oncogenes during the mutation, disrupting the entire process. The tumor suppressor genes mediate uncontrolled cell division (37). DNA methylation, histone modification, and nucleosome position are some of the epigenetic factors playing an important role during cancer (41). The detailed mechanism of cancer at the molecular level has been reviewed (37, 42).

#### Underlying Mechanism of MTX Action in Cancer (Pharmacodynamic)

MTX is considered to be the “targeted” therapy in oncology since its development. Moreover, it was first used in acute lymphoblastic leukemia for its known character of the folate pathway–dependent antimetabolite drug aminoptrein (43). Ironically, it was starting to be used in clinical trials by 1953, but its intracellular targets and DHFR were discovered later (2). This is the first drug used as a single-agent therapy to cure cancer (44) and was used to treat types of cancer, such as leukemia, non-Hodgkin's lymphoma, breast cancer, head and neck cancer, stomach cancer, bladder cancer, bone cancer, and choriocarcinoma (a type of uterine cancer) (2). The oncologic mechanism plays a part in inhibition of purine synthesis, and it stops the cell cycle process in the S phase, subsequently leading to cell apoptosis (7). The mechanism of MTX as a folate antagonist has been considered as a main action in oncology. It acts as an antifolate agent, wherein folates are the building blocks that maintain cell growth (2). The cellular uptake of MTX mediated by the folate receptor group of proteins and their mechanism is described in the later section on RA. MTX mainly blocks the activity of enzyme 5-aminoimidazole-4-carboxamide *ribonu*cleotide (AICAR) transformylase (ATIC) and inhibits the activity of DHFR, an enzyme responsible for catalyzing dihydrofolate (DHF) to tetrahydroflate (THF). The end product of this reaction inhibits the synthesis of thymidylate synthetase (TYMS), which plays a vital role during the synthesis of thymidine residues (7). It has been observed that it reduces the level of both the purine and pyrimidine pool in human T cells together by increasing the level of UTP and decreasing the level of ATP and GTP. It restrains T cell proliferation and enhances apoptosis (7).

#### Nanocarriers for the Effective Delivery of MTX in Cancer Therapeutics

**Figure 1:**
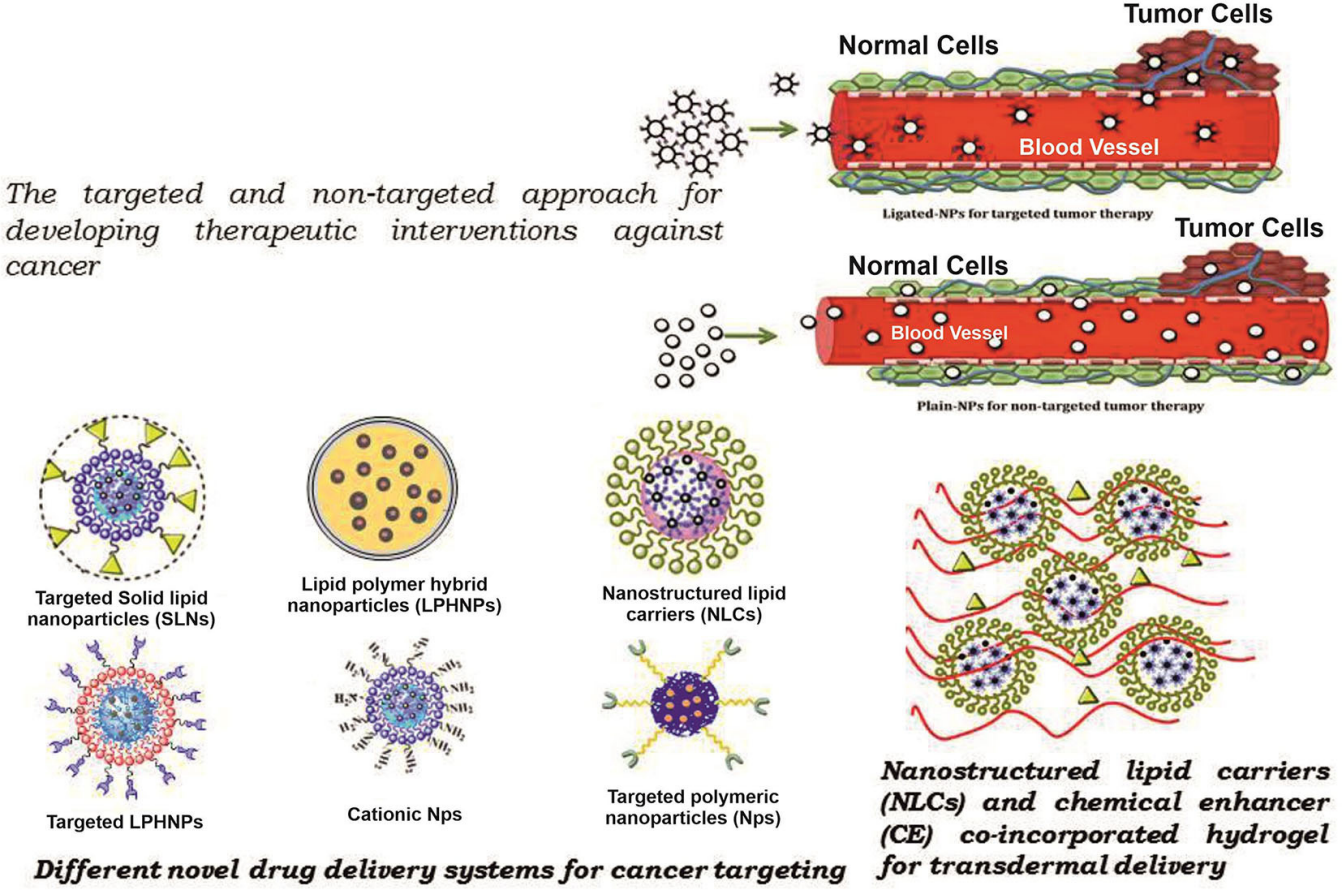
Diagramatic illustration of various potential drug delivery carriers and their structures.

The carrier is one of the most important entities essentially required for the successful transportation of loaded drug(s). The carrier systems are capable of doing so by either inherent or acquired (through structural modification) characteristics to interact selectively with biological targets, or they are engineered to release the drug in close proximity to the target cells *in vitro*, requiring optimal pharmacological action (therapeutic index) (45). Various potential drug delivery carriers and their structure are shown in Figure 1 (46).

##### Polymeric Nanocarriers (PNPs)

Polymeric nanoparticles, polymeric micelles, and dendrimers are the commonly used nanocarriers for the delivery of bioactives (47). PNPs are a type of colloidal drug-delivery system in which the active drug is reduced to the nano-size range (10–1,000 nm), and biodegradable or non-biodegradable polymers are used for the sustained release of the drug (48). Nowadays, biodegradable polymers are used as they are compatible with the body, and no harmful products are formed upon their degradation. Synthetic [polylactic-co-glycolic acid (PLGA), poly(lactic acid) particles (PLA), poly glutamic acid (PGA)] and natural (collagen, chitosan, gelatin) polymers are used in the formulation of PNPs (49). Solvent evaporation, nano-precipitation, emulsification, dialysis, spray drying, salting out, freeze-drying, etc., are commonly used methods for the preparation of PNPs (50).

**Figure 2:**
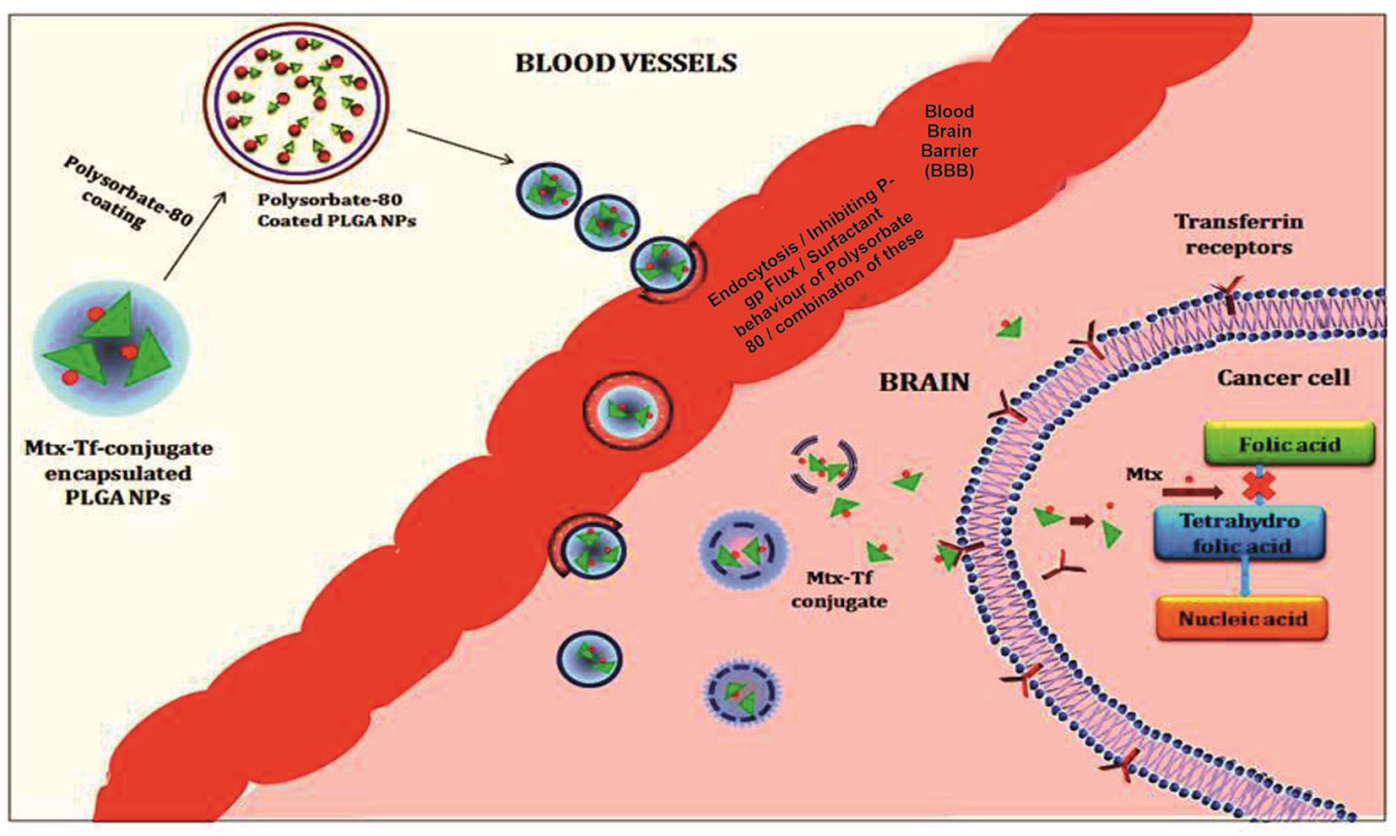
Illustration of the polymeric nanoparticles mediated delivery of methotrexate showing an improved drug efficacy for crossing the blood brain barriers and therapeutic efficacy against brain cancer.

All polymeric nanoparticle–mediated delivery systems for methotrexate that show an improved drug efficacy for crossing the blood–brain barrier and therapeutic efficacy against brain cancer (11) (Figure 2). However, difficulty in scaling up and understudied toxicological studies limits their use and poses challenges to their use as potentially effective novel scale drug carriers (Table 1).

**Table 1:** The advantages and limitations of various nano delivery systems.

**S. no**.	**Nano-delivery systems**	**Advantages**	**Limitation**
1.	Polymeric Nanocarriers	• Improved drug efficacy for crossing the blood–brain barrier • Therapeutic efficacy against brain cancer	• Difficulty in scaling up • Understudied toxicological studies only for lipophilic drugs • Low drug-loading efficiency, Dependency on the concentration of micelle
2.	Dendrimers	• Higher stability • Sustained drug releases	• Poor carrier capacity • Rendered cellular cytotoxicity • Elimination and metabolism depending on the generation and materials and high cost of synthesis
3.	Liposomes	• Surpass the limitation of site-specific oral chemotherapy with the reduced the side effects, • Lower doses need *in vivo* validation	• Require a high production cost • Leakage and fusion of encapsulated drugs • May undergo oxidation and hydrolysis • Shorter half-life and lower solubility
4.	Solid lipid Nanoparticles	• Increase drug stability • Sustained release of drugs • Less toxicity due to the absence of organic solvents, biodegradable, feasible for both kind of drugs • Easy at handling regulatory affairs	• Low drug-loading capacities • Presence of alternative colloidal structures • Complexity of the physical state of the lipid • Possibility of super cooled melts which cause stability issues
5.	Nanostructured Lipid carriers	• Physical stability • Improved drug entrapment and loading efficiencies • Bioavailability and drug release modulation	• Presence of organic solvent residue • Uneven distribution • Complex production process • Poor stability
6.	Lipid polymer hybrid nanoparticles	• Better drug entrapment • Controlled and sustained drug release • Great *in vitro* and *in vivo* stability	

Polymeric micelles are an impressive drug delivery system for poorly water-soluble drugs consisting of hydrophilic polyethylene glycol (PEG) with the particle size range of 10–100 nm, exhibiting EPR and enhanced drug accumulation at the target site (51). Additionally, a computational approach helps in the tailored design of an improved micelles system for multiple drugs in cancer therapy (52). Chen et al. prepared the pluronic-based polymeric mixed micelle (F127/P105-MTX) and compared it with a conventional MTX-loaded polymeric micelle against the overly expressed folate receptor tumor cells *in vitro* (KBv cells) and *in vivo* (KBv tumor-bearing mice). F127/P105-MTX showed the higher (1.36-fold) cellular uptake compared to the conventional conjugate micelle in KBv cells and enhanced antitumor efficacy (53). This result indicates that it could be a possible safe and effective nano-drug delivery system for folate receptor–rich cancer therapy. The hydrophobic core, which is mostly a non-degradable polymer, such as polyacrylamide or polyacrylate, is a concern. Therefore, recently, a bioreducible cross-linked core polymer methoxypoly(ethyleneglycol)-*b-*poly(ε-caprolactone-*co*-α-azido-ε-caprolactone) (mPEG-*b-*poly(ε-CL-*co-*αN_3_εCL)) has been used, and this MTX-loaded core cross-linked micelle was assessed in human breast cancer MCF-7 cells (54). The sustained drug release (76% present inside the cross-linked micelle after 96 h at 37°C in PBS as compared to 90% drug was seen released in the un-cross-linked one) localization at the targeted site (94% uptake without affecting its entry), no toxicity, and significant higher cell death occurred via apoptosis, which make the core cross-linked micelles an emerging and attractive drug delivery system. However, drug release under a reducing environment and further validation through *in vivo* experiments are required (54). Similarly, a novel micelle poly (2-hydroxyethyl methacrylate-Lactide-dimethylaminoethyl methacrylate quaternary ammonium alkyl halide) [P(HEMA-LA-MADQUAT)] was developed for the codelivery of two different anticancer drugs; MTX and chrysin, assessed in MCF-7. Based on cytotoxicity assays, enhanced anticancer activity and suitability as a nanocarrier delivery system showed its importance for use as an anticancer codelivery system for *in vivo* studies followed by clinical trials (55).

To enhance the cellular uptake at the tumor site together with sustained drug release, the novel approach of surface functionalization and changes in the shape of the nanoparticles is proposed as elongated nanoparticles are reported to achieve better drug-delivery efficacy compared to spherical ones (56). Lin et al. used their own previously synthesized PNPs to make functionalized MPEG-PLA-MTX-Cy5.5 nanobacillus by a self-assembly technique in addition to the extrusion-induced transition for local drug delivery at the tumor site (56). The *in vivo* (H_22_ tumor bearing mice) result delineates that intratumorally injected MPEG-PLA-MTX-Cy5.5 showed better target-specific intracellular localization in addition to effective antitumor activity compared to the free MTX and other MPEG-PLA PNP core without drugs. These formulated NPs were employed for conducting *in vivo* fluorescence imaging (56). This novel approach to a delivery system indicates its use for local and tumor-specific cancer therapy without harming the normal tissues, and therefore, it may become a promising delivery system for target-specific cancer treatment. The use of polymeric micelles only for lipophilic drugs, low drug-loading efficiency, and dependency on the concentration of micelle concentration of these carriers are a few obvious limitations. These limitations need to be addressed prior to licensing these carriers for effective delivery of drugs to strategize a treatment strategy against cancer or autoimmune diseases (Table 1).

Dendrimers, are small-sized denritic polymers, a well-organized 3-D structure having a symmetric core and an inner and outer shell, that maintains its structure, density, and function of the surface (57). They have been in use for drug delivery and gene therapy, including other biomedical and translational applications, to study various parameters related to pharmacokinetics and drug delivery systems (58) because they may be used for both hydrophilic and hydrophobic drugs delivered through different routes of administration (59). Kong et al. prepared MTX complexes of classic poly amidoamine (PAMAM) and PEGylated (PAMAM-PEG) dendrimer and administered the formulation in tumor-bearing mice through the intravenous route of administration. The plasma half-lives and mean retention times of MTX complexes were estimated to be higher than MTX with higher antitumor activity (60). Advancement of technologies allowed the deployment of different types of MTX-conjugated dendrimers prepared through various linkages to enhance the antitumor activity (4). Dongen et al. show the binding mechanism of generation 5 (G5) monomer (G5) & dimer (G5-G5) PAMAM-MTX dendrimers with folate-binding protein (61). To address the issue of drug retention, the approach of a dendrimer-conjugated drug with a linker was used by reducing the length of the linkers to make the MTX conjugates (200 Da PEG chain) compared with larger linker conjugates of (GFLG) 450 Da & (GILGVP) 650 Da (62). The smaller length of the linker resulted in less exposure of MTX present in the dendrimer core of PEG and increased bioavailability and transport. These results indicate the potential use of a subcutaneous route for targeted drug delivery specifically for lymphatic sites. Recently, novel dendrimers of MTX (MTX/PGD) were prepared with a co-dendrimer from PAMAM and oligoethylene glycol (OEG) dendrimers to assist in the antitumor efficacy *in vitro* (MCF-7 & 4T1 cells) and *in vivo* (4T1 breast tumor model of BALB/c mice) (63). Significant results in both conditions (cell cytotoxicty IC_50_ after 48 h for MCF-7 and 4T1 was 7.5- and 8-fold higher in MTX/PGD compared to the free MTX) show the potential of MTX/PGD as a promising nanoparticle system with higher stability and sustained drug release due to its highly branched structure and effective biocompatibility (63). Recently, current status in the development of dendrimer-based nanomedicine has been reviewed (64). The poor carrier capacity of dendrimers, rendered cellular cytotoxicity, elimination and metabolism depending on the generation and materials, and high cost of synthesis are obvious limitations of dendrimers (Table 1).

##### Liposomes

Liposomes are artificially prepared spherical vesicles that are composed of a phospholipid bilayer in which cholesterol is usually added to confer stability to the lipid bilayer for optimum drug release (65). Liposomes are composed of one or more lipid bilayers and categorized as small unilamellar vesicles (SUV), large unilamellar vesicles (LUV), and multilamellar vesicles (MLV) (66) based on their size and number. They are versatile as they may deliver both hydrophilic and lipophilic therapeutic agents (67, 68). Moreover, targeting can be achieved by anchoring ligands on the surface of the liposomes that are specific to a particular cell type. The choice of phospholipids used during the preparation of liposomes largely depends upon the desired rigidity and permeability (65, 69).

The poor water solubility of MTX and good lipophilic properties of liposomes establish their use for its effective delivery (3, 4). Despite the advantages of the liposomal drug delivery system, the foremost goal of NDDS is to improve the bioavailability of the therapeutic agents and reduce the side effects by enhancing the pharmacokinetic and pharmacodynamic properties (68).

The interest in local targeted drug delivery systems gained attention as muco-adhesive patches of MTX-loaded liposomes (MTX-L) were prepared by the thin film hydration method using phosphatidylcholines (PC) and cholesterol (70) for targeted delivery in oral cancer to circumvent side effects of conventional methods, including chemotherapy, radiation, and surgical excision. MTX-L was cast in muco-adhesive film by using different polymers, such as hydroxyethyl cellulose (HEC), PVA, PEG, and chitosan (CH), and assessed for their parameters, including thickness, weight, percentage swelling index, sustained drug release, and pattern (70). An *in vitro* cell viability assay confirmed the significant cell death measured by IC_50_ was 180 μg/mL (free MTX) and 75 μg/mL (MTX-LP-F7, different mucoadhesive buccal films with different concentrations of polymer). In conclusion, the liposomes prepared for oral delivery by using different polymers may have the advantage for sustained drug release with increased bioavailability. The MTX patches can surpass the limitation of site-specific oral chemotherapy with the reduced side effects and lower doses needing *in vivo* validation. Of late, the role of surface functionalization and different targeting strategies for the liposomal drug delivery system in solid tumors have been extensively studied (71). Despite the abovementioned advantages, following are the limitations of liposomes as nanodrug delivery vehicles (Table 1):

- Liposome-encapsulated drugs require a high production cost.
- Liposomes may have leakage and fusion of encapsulated drugs.
- The liposome phospholipid may undergo oxidation and hydrolysis.
- Liposomes have a shorter half-life and lower solubility.

##### Solid Lipid Nanoparticles (SLNs)

The different issues related to drug delivery, regulatory affairs, and the availability of cheaper liposomal formulation are some of the concerns related to the development of an advanced nanoparticle, resulting in the formulation of first-generation nanoparticles. These nanoparticles prepared using solid phase lipids and surfactants are famously termed “solid lipid nanoparticles” (72). The solid lipid remains in its intact form at body and ambient temperatures, whereas surfactants are used as an emulsifier in the range of 0.5–5% to confer stability (72, 73). Among the methods used for the preparation of SLNs, commonly used methods are high-pressure homogenization; high-shear homogenization; and ultrasound, hot/cold homogenization, solvent evaporation, spray drying and emulsification (72, 74). The drug load in SLNs is dependent upon the solubility of the drug molecule in the lipid, the polymorphic state of the drug molecule, and the properties of the solid lipid matrix (73). SLNs may be administered by different routes, such as oral, parenteral, nasal, ocular, and transdermal. The release of the drug from SLNs is inversely related to the partition coefficient of the drug and its crystalline behavior (74). Based on the preparation method, SLNs are divided into three main types: (1) solid solution, (2) drug-enriched shell, and (3) drug-enriched core (74). These NPs have the advantages of drug stability, sustained release of drugs, less toxicity due to the absence of organic solvents, biodegradability, feasibility for both kind of drugs, and easy handling of regulatory affairs (72, 74, 75). Solid lipid nanoparticles have many potential applications in developing therapeutic interventional approaches against cancer chemotherapy, brain targeting, parasitic diseases, tuberculosis, gene delivery, and in dermatological preparations (72, 73, 75).

The overexpression of lectin receptors on cancerous cells was used as a target, and MTX-SLNs were prepared by the hot microemulsion method followed by fucose coating (MTX-SLN-F). The *in vitro* cell cytotoxicity (MCF-7 cells) was reported (IC_50_) to be higher in MTX-SLN-F (~2 μg/mL) compared to MTX-SLN (~3 μg/mL) and free MTX (~7 μg/mL) after 72 h with an increased cell uptake of ~70% after 3 h in the case of MTX-SLN-F compared to ~45 and ~10% for MTX-SLN and free MTX, respectively (14). The *in vivo* antitumor effects of MTX-SLN-F and free intravenous MTX injection were observed in breast cancer–bearing female Wistar rats and the percentage of tumor burden was significantly high (*P* < 0.001): ~ 30, 53.8, and 62% by MTX-SLN-F, MTX-SLN, and free MTX after 4 weeks of treatment, respectively. There was no mortality found after several rounds of injection in the case of MTX-SLN-F compared to 66.66 and 50% of mortality observed in free MTX and MTX-SLN formulation 10 weeks posttreatment (14). Results of the experiments favored the approach of preparing a ligand-anchored SLN-encapsulated drug for breast cancer therapy. Recently, Battaglia et al. prepared MTX-SLN by using the method called “coacervation” (76): a solvent-free method wherein fatty acid precipitation was seen due to the reduction of pH by acidification of micellar solutions in the presence of a polymeric stabilizer (77). They used the prepared SLNs against a glioblastoma multiforme (GBM) stage IV glioma. Didoceylmethotrexate (ddMTX), a lipophilic MTX ester, is used as MTX has issues with entrapment. The *in vitro* cytotoxicity against rat F98 cells was found to be encouraging (however, not significant) and provided some preliminary data for GBM (77). All above-given accounts suggest an advantage of SLNs over traditional drug-delivery systems for poorly water-soluble drugs. It is advised that SLNs be used as efficient delivery systems. However, there are some constraints in using these SLNs, and they include low drug-loading capacities, presence of alternative colloidal structures (micelles, liposomes, mixed micelles, drug nanocrystals), the complexity of the physical state of the lipid (transformation between different modifications), and the possibility of super-cooled melts, which cause stability issues (Table 1).

##### Nanostructured Lipid Carriers (NLCs)

Second-generation solid lipid nanoparticles called “nanostructured lipid carriers” (NLCs) are formulated to address the existing limitations of SLNs (72, 74). NLCs are the most preferred nanodrug delivery system nowadays among NDDS due to their advantage over others with respect to physical stability, improved drug entrapment and loading efficiencies, bioavailability, and drug release modulation (78). NLCs are colloidal lipid nanoparticles prepared by mixing solid and liquid lipids together with surfactants (as an emulsifier). Most of the drugs are fairly soluble in liquid lipids compared to solid lipids; thus, the drug leaching observed with SLNs is overcome by the entrapment of the drug (72, 79). During the preparation and storage of NLCs, the formulation goes through lower temperatures during homogenization and crystallization. The cooling process decreases the solubility of the drug in the lipophilic phase, therefore, showing drug expulsion from the nanoparticles, especially with the use of higher concentration drug formulations (78–80). A drug can be encapsulated in the space between the solid lipid molecules in a crystalline structure that is created due to the imperfections in the organization of the crystal. Thus, the higher the disturbance in a crystalline structure, the more the drug is encapsulated (72, 74, 79, 80). For achieving maximum stability, the recrystallization of the lipid in the cooling process is reduced and retarded in the case of NLCs compared to the extent seen in SLNs because the crystal order is highly disturbed because of oil particles remaining in the liquid phase in NLCs (74, 79). The engineering of NLCs is aimed at increasing drug loading of therapeutic agents and preventing the leakage of drugs upon storage (79, 81, 82). High-pressure homogenization, hot/cold homogenization, solvent evaporation, emulsification, solvent diffusion, solvent emulsification-evaporation, phase inversion, solvent injection/displacement method, etc., are some of the commonly employed methods for the preparation of NLCs (74, 79, 81). Different types of liquid lipids—mainly soybean oil; medium chain triglycerides/caprylic- and capric-triglycerides; oleic acid; corn oil; and solid lipids, such as stearic acid, glyceryl monostearate, cetyl palmitate, glyceryl palmitostearate, glyceryl behenate, grades of witeposl® and softisan®–are used in the preparation of NLCs (81, 83). Surfactants (Tween 80, lecithin, poloxamer 188, Polyglyceryl-3-methylglucose distearat, sodium dodecyl sulfate, sodium deoxycholate, Tween 20, Myverol^TM^ 18-04K, PVA, solutol® HS 15 and polyoxyl castor oil) are used during the preparation of NLCs to provide stability to the formulation. Drug-loaded NLCs are administered by oral, topical, parenteral, and ocular routes to address brain-related issues (72, 78, 80, 81, 83). In cancer therapy, two important factors, namely real-time monitoring or diagnosis and treatment of affected tissues, play a central role. By focusing on these aspects, Kohler et al. formulated magnetic nanoparticles using a modified coprecipitation method (84). Magnetite, Fe_3_O_4_, nanoparticles were first surface-modified with (3-aminopropyl) trimethoxysilane (APS) to make the self-assembled monolayer followed by amidation between carboxylic acid end groups on MTX and an amide group present on the surface of the particle. Prepared MTX-NLCs were studied in MCF-7 and human cervical cancer cells (HeLa) for drug efficacy and drug release to determine the cell uptake (84). Cell viability after 120 h was found to be similar to free MTX. The cell uptake via MTX-NLCsbyMCF-7 (20 times) and HeLa (10 times) was found to be higher. TEM image analysis after the internalization of MTX-NLCs confirms the release of MTX within the lysosomal compartment (84).

The preparation strategies of NLCs, considering selection of solid and liquid lipids, type of surfactants and their formulation, play an instrumental role in biomedical applications in cancer therapy (85) and autoimmune systemic inflammatory diseases, such as psoriasis and RA (86). At present, research on NLCs is limited to preclinical studies with clinical applications remaining far from realization. There are some limitations, such as the presence of organic solvent residue, uneven distribution, complex production process, and poor stability (Table 1).

##### Lipid Polymer Hybrid Nanoparticles (LPHNPs)

The low solubility, dose-related toxicity, non-specificity, rapid diffusion throughout the body, short half-life in the bloodstream, and development of drug-resistance of conventional lipid- and polymer-based nanoparticles by the target cell (87) are some of the glaring issues with the existing drug-delivery vehicles. The innovative NDDS lipid–polymer hybrid nanoparticles (LPHNPs) combine the attributes of polymeric and lipid nanoparticles (PNPs) (46, 87–89). Lipophilic and poorly water-soluble drugs can be incorporated in the hydrophobic core of the polymer. They are prepared by two methods: (1) two-step (conventional and non-conventional) and (2) one-step (by nano-precipitation and emulsification-solvent-evaporation) (89, 90). LPHNPs offer a versatile drug-delivery system with better drug entrapment, controlled and sustained drug release, great *in vitro* and *in vivo* stability (89). In addition, the lipid layer slows down the rate of polymer degradation of LPHNP products by limiting inward water diffusion and helps the sustained-release kinetics of loaded content (89). The properties of LPHNPs advocate for their utility and prove advantageous over existing delivery vehicles (87, 91). Thus, well-designed LPHNPs contain hydrophobic polymeric core functions, whereas the surrounding lipid coat is a biocompatible shield and a barrier preventing the fast leakage of water-soluble drugs (92, 93). Properties such as biocompatibility, biodegradability, sustained drug-release profiles, and greater loading capacity are attributed to a stable, high-payload, targeted drug-delivery system that might maximize chemotherapeutic efficacy against targeted cancer cells (90, 94). Recently, self-assembled polymer-lipid hybrid NPs were developed aiming at overcoming the limitations seen with conventional drug-delivery systems. The polymer-lipid hybrid NPs gained significant attention for drug and gene delivery (95, 96). LPHNPs were engineered with an intent to explore the characteristics of lipid and polymeric nanoparticles in one delivery system (89, 91) to achieve controlled and targeted drug delivery for the treatment of cancer and other inflammatory diseases (90, 97). Therefore, Tahir et al. used different concentrations of polymer (PLGA), lipid (Lipoid S100), and surfactant (Lutrol® F-68) for the preparation of MTX-LPHNPs by the single-step, self-assembly, modified nano-precipitation method to check the influence of variation on particle size, entrapment efficiency (EE), and drug release using a three-level box Behnken design (Design-Expert®software) (98). Particle size range was increased with polymer concentration, whereas EE was dependent on both the lipid and polymer concentrations. The antiproliferative activity against the PC3 and MDA-MB-231 cells by the ATP activity–based assay showed higher growth inhibition even at the highest concentration (200 μg/mL) when MTX was encapsulated in LPHNPs compared to free MTX (98).

The surface of NPs is conjugated with the targeting molecules, which are recognized by the receptors expressed by the ailing cells (99) to achieve better targeting efficiency and offer novel and much better cancer therapeutic approaches (100–103). Further, active targeting of LPHNPs increases the probability of drug availability at the target site, which eventually reduces the chances of exposure of healthy cells and reduces adverse effects (104). The overly expressed membrane receptors (lectin receptors/LRs) are exploited by the drug targets during tumor pathogenesis (105–108). The presence of lectin (carbohydrate) moieties on the surface of therapeutic NPs can efficiently enhance specificity and binding affinity, eventually leading to significantly higher cellular uptake through receptor-mediated endocytosis (105, 107–111). The lectin receptor-mediated targeting employs the interaction of endogenous ligands with different sugar moieties, such as galactose (G), mannose (M), fucose (F), fructose, and lactose (109–112). This nano-carrier system results in glycosylated carriers comprising carbohydrate as stratum ligands, which are known for quick internalization through lectin receptor-mediated endocytosis (105–108).

MTX-acelofenac (ACE)-loaded LPHNPs were prepared by a single-step, self-assembled, nano-precipitation method to achieve codelivery of MTX and ACE in fucose-anchored LPHNP (LPHNP-MTX+ACL-Fu) approaches against breast cancer cells (MCF-7 and MDA-MB-231) (88). The immediate localization (within 2 h of incubation), enhanced bioavailability (8–10 times higher than free drugs), and higher cell cytotoxicity (increased cell death of MTX during coencapsulation with ACL compare to free drugs) was observed in the MTT assays conducted *in vitro* (88). The *in vivo* experiments were carried out in DMBA-induced cancer BALB/c mice. The pharmacokinetics (mean residence time 5–6 times higher than free MTX and ACL), sustained drug release (measured up to 72 h when administered intravenously), and antitumor activity (residual tumor burden 19.54%, 33.73%, and 163.8% for LPHNP-MTX+ACl-Fu, LPHNP-MTX+ACl, and normal saline-untreated control for 5 weeks, respectively) (88) confirmed the synergistic effects as evaluated by the pharmacological parameters, conferred by the codelivery of drugs. Later on, fructose-tethered MTX and beta carotene (BC)-loaded LPHNPs (F-MTX+BC-LPHNPs) were engineered by the self-assembled nano-precipitation method for the treatment of breast cancer to find out the role of BC on MTX-mediated cytotoxicity (113). F-MTX+BC-LPHNPs showed a high apoptosis index (0.89) in MCF-7 cells and sustained drug release in a biphasic manner up to 120 h for F-MTX+BC-LPHNPs and MTX+BC-LPHNPs, resulting in improved bioavailability with enhanced localization at the tumor site. Similarly, female Wistar rats bearing cancer induced by DMBA were injected with different formulation's repeated intravenous administration (once in 3 days) [114]. The tumor progression was measured 30 days posttreatment and found to be 32% in F-MTX+BC-LPHNPs compared to 43.2 and 63.1% with MTX+BC-LPHNPs and free MTX, respectively. Moreover, β-carotene helps in the refinement of renal and hepatic toxicity [114] when mixed in the formulations. Results uncover the potential use of bioactives in the future with LPHNPs for targeted and sustained drug delivery for various treatments of cancer, autoimmune diseases such as RA, and psoriasis.

We have, as have others (1, 90), recently reviewed the current status and future application of LPHNPs in details for cancer therapeutics (46).

## Concluding Remarks and Future Directions

Various studies demonstrate MTX as a revolutionary medicine in the field of biomedical sciences as it shows significant therapeutic potential, selective targeting, robust biological response, and ensured safety. In addition, the residence time of MTX in NLCs is extended in blood circulation and, thus, permits MTX to accumulate at the desired sites. There is lots of research going on focusing on combinatorial cancer therapy and in inflammatory disorders, and it is likely to expand in the future. Moreover, the MTX platform may be utilized for multiple activities simultaneously with imaging and drug-delivery characteristics. They may have multiple applications in cancer chemotherapy and other clinical settings.

## Author Contributions

W-JY conceived the idea and prepared, edited, and finalized the manuscript. D-XH and SL helped write the manuscript. Y-LS rendered all chemicals and helped write the manuscript. FG and HL provided reagents and helped prepare the manuscript. All authors contributed to the article and approved the submitted version.

## Conflict of Interest

The authors declare that the research was conducted in the absence of any commercial or financial relationships that could be construed as a potential conflict of interest.

## Abbreviations

MTX: Methotrexate
MW: Molecular weight
RA: Rheumatoid arthritis
DHFR: Dihydrofolatereductase
NDDS: Novel drug delivery systems
NPs: Polymeric nanoparticles
LPHNPs: Lipid polymer hybrid nanoparticles
NLCs: Nanostructured lipid carrier
SLNs: Solid lipid nanoparticles
HPLC: High performance liquid chromatography
FPI: Fluorescence polarization immunoassay
IV: Intravenous
SC: subcutaneous
IM: Intramuscular
CNS: Central nervous system
CSF: Cerebrospinal fluid
PLGA: Polylactic-co-glycolic acid
PLA: Poly lactic acid
PGA: Poly glumatic acid
PEG: Polyethylene glycol
EPR: Enhanced permeability and retention time
ATIC: 5-aminoimidazole-4-carboxamide ribonucleotide (AICAR) transformylase
PC: Phosphatidylcholine
AUC: Area under the curve
EE: Entrapment efficiency
GVHD: Graft versus host disease
aGVHD: acute graft versus host disease
cGVHD: Chronic graft versus host disease
ACE: Acelofenac
MTT: 3-(4,5-Dimethylthiazol-2-yl)-2,5-Diphenyltetrazolium Bromide
BC: Beta carotene
DMBA: 7,12-Dimethylbenzathracene
RF: Rheumatoid factor
ACAP: Anti-citrullinated peptide antibodies
AUR: American college of Rheumatology
EULAR: European League Against Rheumatism
csDMARD: Conventional synthetic diseases-modifying antirheumatic drugs
bDMARD: Biological DMARD
HA: Hyaluronic acid
FA: Folic acid
AIA: Adjuvant induced arthritis
siRNA: Small interfering RNA
APCs: Antigen presenting cells
LFA: Lymphocyte functional antigen
Th cells: T helper cells
PASI 75: Psoriasis area and severity index
SUV: Small unilamellar vesicles
LUV: Large unilamellar vesicles
MLV: Multilammellar vesicles
OA: Oleic acid
NIPAM: N-isopropylacrylamide
PEG_2_: Prostaglandin E2
GFLG: Glycine-phenylalanine-leucine-glycine
GILGVP: Glycine-isoleucine-leucine-glycine-valine-proline
HCT: Hematopoietic cell transplantation
BSA: Body surface area
GM-CSF: Granulocyte-macrophage colony-stimulating factor
ABCC: ATP-binding cassette proteins.
